# Clinical and Pathophysiological Significance of Trace Elements in Oral Potentially Malignant Disorders and Oral Cancer: A 20‐Year Narrative Review for Evidence‐Based Public Health Intervention

**DOI:** 10.1155/ijod/3886827

**Published:** 2026-09-02

**Authors:** Hannah Haneef, Ravikiran Ongole, Joanna Baptist, Sara J. Ommen, Prasanna Mithra

**Affiliations:** ^1^ Oral Medicine and Radiology, Manipal College of Dental Sciences Mangalore, Manipal Academy of Higher Education, Manipal, India, manipal.edu; ^2^ Oral and Maxillofacial Surgery, Manipal College of Dental Sciences Mangalore, Manipal Academy of Higher Education, Manipal, India, manipal.edu; ^3^ Community Medicine, Kasturba Medical College Mangalore, Manipal Academy of Higher Education, Manipal, India, manipal.edu

**Keywords:** elemental analysis, good health and well-being, oral cancer, oral potentially malignant disorders, trace elements

## Abstract

Our body is made up of many elements and compounds that work together to keep us healthy. Among them are trace elements, which are needed only in tiny amounts but play a very large role in protecting our cells from damage. When these trace elements are too low or too high, they can disturb the body’s balance and may increase the risk of diseases such as oral cancer. In this study, we investigated how different trace elements are linked to early signs of oral cancer and precancerous conditions over the past 20 years. We reviewed studies available in the PubMed database that used elemental analysis to detect changes in oral tissues before cancer fully develops. Understanding how these trace elements affect oral health can help us shape better prevention policies. If we know which elements people in certain areas lack or have in excess, health authorities can design nutrition‐based programs, awareness campaigns, and screening strategies to prevent oral cancer early. Linking science with public health policy can strengthen preventive oral care and help build a future where fewer people develop oral cancer.

## 1. Introduction

The human body is composed of various elements and compounds. Water and minerals form inorganic compounds, whereas fat, carbohydrates, proteins, and nucleic acids form organic compounds. Most of these compounds contain elements. In a description by Heymsfield et al., the human body can be organized into five levels, namely, atomic, molecular, cellular, tissue systems, and the whole body. Ninety‐nine percent of the human body consists of just six elements: hydrogen, oxygen, carbon, nitrogen, phosphorus, and calcium. Approximately 0.85% of the remaining mass is made up of another five elements, mainly sulfur, potassium, sodium, chlorine, and magnesium. Together, these 11 elements are considered essential. A total of 0.15% of the remaining elements are composed of trace elements. Many of these trace elements function primarily as catalysts in various enzymatic reactions. The human body contains trace elements in minute quantities. The term “trace elements” refers to substances that are found in trace concentrations in both natural and disturbed settings, with excessive bioavailability having a harmful effect on living things [1]. Deficits in any of the trace elements are prevented by robust homeostatic systems, and trace elements are essential to many processes required for life. When an essential trace element is either lacking or present in insufficient quantities, it might have life‑threatening implications. The processes of absorption, storage, and excretion are involved in homeostatic control. The importance of these three processes significantly differs among each trace element [2].

## 2. Methodology

This study did not require Institutional Ethics Committee approval as it was a narrative review.

### 2.1. Search Strategy and Data Sources

A systematic electronic literature search was conducted primarily via the PubMed, Scopus, and Cochrane Library databases to identify original articles, case reports, and comparative studies related to the influence of trace elements on the early diagnosis and etiopathology of potentially malignant oral disorders and oral cancer. The search was strictly limited to articles published from January 2006 to December 2023 to capture the most current and relevant evidence. The query employed a combination of Medical Subject Headings (MeSH) terms and keywords, utilizing the Boolean operators AND and OR to maximize both sensitivity and specificity.

The comprehensive search query was as follows: (“Trace Elements” OR “Micronutrients”) AND (“Oral Precancerous Conditions” OR “Oral Potentially Malignant Disorders” OR “Oral Cancer”) AND (“Elemental Analysis” OR “Spectrometry”).

### 2.2. Selection and Data Extraction

Articles were selected if they were original research (including case reports or comparative studies), focused on the link between trace elements and oral precancerous conditions (e.g., leukoplakia (L), oral submucous fibrosis [OSMF], and oral lichen planus [OLP]) or oral cancer, and employed elemental analysis techniques (e.g., atomic absorption spectroscopy [AAS], energy dispersive X‐ray spectroscopy [EDX], and mass absorption spectrometry [MAS]) to measure trace element levels in serum, tissue, or saliva. Articles were excluded if they were reviews, editorials, or conference abstracts without full data; if they did not focus on the oral cavity; or if elemental analysis was used as a primary investigative method.

The search results from each database were exported to a common platform, the Mendeley software, and subsequent deduplication was performed. The authors undertook a full‐text review of the studies finalized for the review and identified themes relevant to the research objective.

### 2.3. Data Synthesis

A narrative approach was conducted as per the established guidelines to synthesize the findings.

The final keywords used for indexing this review were trace elements, oral potentially malignant disorders (OPMDs), oral cancer, elemental analysis, and good health and well‐being (aligned with the public health policy context).

## 3. Results

### 3.1. Role of Trace Elements in OPMDs

Extensive research conducted recently on the influence of trace elements on the etiopathology of precancer or cancer has revealed interesting findings. Early diagnosis is possible through the detection of biochemical and immunological changes in the patient’s serum, and as the disease progresses, trace elements act as prognostic indicators, enabling suitable treatment for the patient [3].

### 3.2. Oral L (OLK)

L stands out as the most prevalent potentially malignant oral lesion, showing increased deviation from malignancy. Recently, there has been a growing focus on trace elements in the identification of oral cancer and precancer due to their notable alterations in head and neck carcinomas. In the development and progression of carcinogenesis, the copper (Cu)‐to‐zinc (Zn) ratio has emerged as a reliable biomarker. The cause of L is unknown, and its etiopathogenesis is complex. Among the risk factors that are observed are the use of tobacco in smoked or smokeless forms, which is the most common. Cu has attracted significant attention as the most widely researched trace element in precancerous and cancerous conditions, with these serum elements proving to be reliable diagnostic and prognostic indicators. Alterations in Cu metabolism are notable in neoplastic diseases, with the serum Cu concentration correlated with malignant progression, tumor incidence, recurrence, and burden across various cancers. Cu plays a pivotal role in tumor angiogenesis, particularly in the initial stages. It is essential for endothelial cell activation, stimulating the proliferation and activation of these cells. Cu triggers various angiogenic factors that induce cells in the endothelium to transition from the G0 phase to the G1 phase and promote their multiplication [4]. Moreover, elevated serum levels of Cu contribute to the production of increased levels of free radicals through metal‐catalyzed Haber–Weiss reactions, potentially playing a role in the carcinogenic processes associated with tobacco and areca nut usage [5].

Mampilly et al. [6] evaluated the serum selenium (Se) and serum ceruloplasmin concentrations in blood samples from patients with OLK and oral squamous cell carcinoma. A total of twenty patients and ten healthy persons between the ages of thirty and sixty were selected. Three groups were formed out of the subjects: ten healthy people made up Group 1, ten patients with OLK (10 patients in Group 2), and ten patients with squamous cell carcinoma (SCC) (10 patients in Group 3). Blood was selected as the sample for investigation. Ceruloplasmin was measured by the diamine oxidase method. The method of atomic absorption developed by Sir Alan Walsh in 1950 has become the preferred method for the elemental analysis of Se (atomic absorption spectrometer). A comparison of the levels of ceruloplasmin between the groups indicated that the average value in Group 1 and the control group was 31.746 mg/dL, the average value in Group 2 with L was 81.411 mg/dL, and the average value in Group 3 with SCC was 90.7120 mg/dL. When the Se levels in each group were compared, Group 1 had an average value of 119.937 ng/mL, Group 2 had an average value of 109.17 ng/mL due to L, and Group 3 had an average value of 99.6230 ng/mL due to SCC. Patients with L may benefit from using serum levels of Se and ceruloplasmin as disease markers. Due to Se’s preventive function as an antioxidant against cancer, patients with L and oral squamous cell carcinoma may have lower serum Se concentrations. Serum ceruloplasmin typically has a normal range of 20–60 mg/dL. The findings demonstrated elevated levels of ceruloplasmin in individuals with SCC (average of 90.7120) and L (81.4110) as compared to healthy people (31.7460) [6]. As a study of L specifically, this paper raises a hypothesis worth testing, ceruloplasmin as an early premalignant marker, but provides essentially no evidence capable of distinguishing that hypothesis from simpler explanations like tobacco habit or inflammation from the existing oral lesions.

The striking feature is that the L group’s ceruloplasmin (81.411 mg/dL) is much closer to the SCC group’s value (90.712 mg/dL) than to the control value, implying that ceruloplasmin elevation occurs early, at the premalignant stage, almost to the same magnitude as in carcinoma. Se, by contrast, shows a more graded, intermediate decline in L between control and SCC values. These two patterns deserve separate scrutiny because they imply different biological risks.

Sourabh et al. [7] conducted a study to correlate the amounts of the trace elements iron (Fe), Cu, and Zn in the serum between patients with oral squamous cell carcinoma, patients with L, and healthy individuals. Eighty patients were included in the study: Thirty had L, thirty had oral squamous cell carcinoma, and twenty were in the control group, indicating that they had no relevant medical, dental, or habit history. Using an anticubical vein puncture, 10 mL of peripheral blood samples from individuals with oral squamous cell carcinoma and L as well as control individuals was obtained. Without the use of any chemicals or anticoagulants, the blood was drawn into a simple red tip vein puncture tube and allowed to clot naturally at room temperature, and the serum was separated from the cells via centrifugation at 4°C and 3000 revolutions per minute. Prior to analysis, the separated sera were stored at –20°C. The levels of Cu and Zn in the serum were measured via AAS. In the present study, an atomic absorption spectrophotometer was used to assess the levels of Zn and Cu. A RANDOX kit was used to estimate serum Fe levels (Siedel, 1984). Serum trace element analysis offers an economical, noninvasive method for the detection, diagnosis, and surveillance of malignant lesions such as oral squamous cell carcinoma as well as premalignant L lesions [7]. The Cu‐to‐Zn (Cu/Zn) ratio does not appear to be reported in this study’s given results. The study does not establish whether trace element alterations precede L (causative) or result from it (consequence).

A study by Bose et al. [8] on patients with L estimated the antioxidant vitamins in plasma, the antioxidant minerals Zn and glutathione, and the total antioxidant status (TAS) concentration. This study examined 23 diagnosed OLK patients who were 28–40 years of age and of both genders and healthy individuals who were of the same age and sex‐matched without any history of systemic ailments as the control group. They assessed the plasma levels of the antioxidant vitamins C, A, and E; the antioxidant metabolite GSH; the antioxidant mineral Zn; and the TAS of the groups. In L patients, the observed plasma Zn concentration was relatively low, possibly because of the utilization of Zn to counteract the reactive oxygen species (ROS) generated by tobacco or because of the increased Cu content in the metabolism of areca quid [8]. The authors themselves note that tobacco and areca quid contain high Cu content, which is relevant to the high dietary/oral Cu exposure that can antagonize Zn absorption and lower plasma Zn independent of any premalignant pathology, making this a likely contributing factor.

The study provided a single average Zn value for L cases, without considering differences in dysplasia severity or lesion characteristics. This means the reported value tells us only that Zn is low in some average L patients—it cannot answer the clinically far more important question of whether Zn depletion is deeper in higher‐grade lesions, which would be required to support Zn as a marker of malignant transformation risk.

The authors themselves acknowledge that the consumption of tobacco or areca quid generates high levels of ROS during metabolism. However, the methods section does not report habit type, frequency, duration, or quantity for either the L or the control group. This is a critical factor for Zn finding specifically.

Studies have shown that the metabolism of Cu significantly changes within various conditions, which is associated with the presence of a tumor, its load, advancement, and occurrence in various human cancers and with serum Cu levels. Cu plays a role in the initial stages of tumor angiogenesis. Zn is necessary for regulating the cell cycle and division and for the activity of DNA polymerase and is especially necessary for the rapid increase in the number of cells owing to tumor growth [9].

### 3.3. Oral Submucous Fibrosis

Cu is crucial as a cofactor for enabling lysyl oxidase enzyme activity, and elevated levels of Cu in areca nuts significantly influence the development of OSMF. They initiate fibrogenesis by increasing lysyl oxidase activity and restricting collagen degradation. The increased production of Cu‐containing ceruloplasmin results in increased serum Cu levels by the liver as a part of the inflammatory response or from a reduced breakdown in serum ceruloplasmin levels [10]. Because one of the biochemical indicators for nutritional assessment is serum Fe levels, any depletion in their value may suggest that the oral precancer conditions have progressed to a stage of malignancy. Inadequate nutrition may lead to deficiencies in Fe and folic acid and vitamin B12, which may harm the oral mucosa, thereby resulting in reduced levels of serum Fe [11]. Zn, as an element, ensures the stability of biological membranes and plays a vital role in the functioning of immune cells, defense against antioxidants, and adequate wound healing. The possible damage to nucleic acids and proteins in the body results from the generation of ROS by the act of chewing areca nuts. The formation of ROS is suppressed when the activation of the antioxidant enzyme superoxide dismutase (SOD) is triggered by Zn [12]. Zn helps in mucosal metallothionein regulation and thereby reveals an inverse relationship with Cu, which affects Cu absorption [13]. Cancer cells possess uncontrolled Zn importers, leading to elevated Zn levels that increase the survival of immune cells and induce tumor cell death. Se is an important component of various enzymes, including glutathione peroxidase, fatty acid binding protein, metalloprotein, selenoprotein P, deiodinase, and type I iodothyronine. Excess amounts of Se can be poisonous, but small traces are vital for cellular functions. Additionally, Se is considered a nutritional antioxidant, and decreased levels of Se in the serum, plasma, or blood are associated with the risk of developing malignant lesions in various areas, such as the esophagus, colon, prostate oral cavity, breast, and ovary [14]. According to estimates, the normal intake of Se ranges from ~20 to 70 µg/day [15].

A study by Trivedy et al. [16] assessed whether there are increased Cu and serum levels in individuals diagnosed with oral submucosal fibrosis due to the consumption of areca nuts. The concentrations of Cu in the tissue were analyzed by collecting a few biopsies of the buccal mucosa from individuals with oral submucosal fibrosis in Nagpur, India, and were assessed by MAS. MAS revealed that the average tissue concentration of Cu was 5.5 μg/g in the oral submucosal fibrosis samples, whereas it was 4 μg/g in the nonareca chewing controls. The presence and diffusion of the metal were estimated via energy dispersive X‐ray microanalysis. Clear peaks suggestive of Cu in the epithelium (21 of 23) and connective tissue (17 out of 23) of the oral submucosal fibrosis samples, in contrast with spectra, were obtained from oral biopsies of controls of nonareca chewing subjects. The ceruloplasmin (0.32 g/L), serum Cu (17.23 μmol/l), and urinary Cu (0.52 ± 0.26 μmol/l) levels in oral submucosal fibrosis patients were within the normal range. The hypothesis of this study is that the incidence of Cu in oral submucosal fibrosis tissue is an instigating factor in oral submucous fibrosis, potentially contributing to fibrogenesis by increasing lysyl oxidase activity. Compared with those of other commonly consumed nuts, the content of Cu was found to be greater in areca nuts, and the levels of dissolved Cu increased in mouth fluids when nuts were chewed for 5–30 min [16]. The study does not extensively elaborate on any other trace elements (such as Zn, Fe, or Se) that may interact with Cu metabolism or independently contribute to fibrogenesis. This represents a significant limitation in scope, as OSF is likely an alteration in several trace elements rather than a single‐element phenomenon.

Although the possible connection between Cu and areca nut use is discussed, the study does not quantify the duration of habit, frequency of chewing per day, or the type of areca nut preparation used among OSF patients. These variables could significantly influence the quantity of Cu absorbed into oral tissues and thus the measured tissue Cu levels.

The study does not explore other trace elements. Cu does not function independently in biological systems—it competes with Zn for intestinal absorption and shares enzymatic pathways. The lack of Zn, Fe, and manganese (Mn) measurements limits the understanding of overall trace element changes in OSF.

In chronic inflammatory conditions where oxidative stress intensifies, the increase in Fe levels in OSF tissue is highly important. Fe and Cu play interdependent roles in maintaining stability at the tissue elemental concentration level. A notable reduction in Cu within OSF triggers pathophysiologic situations conducive to Fe‐mediated free‐radical formation, thereby exacerbating inflammation and promoting the progression of fibrosis‐associated collagen synthesis [17].

In an investigation by Nayak et al. [18], individuals residing in the coastal area of southern Karnataka along with Kerala, India, consuming areca nuts were evaluated for the concentrations of these trace elements. The study involved 20 individuals diagnosed with oral submucosal fibrosis and 20 control subjects. Group A comprised individuals diagnosed clinically with OSMFs, and group B comprised participants without any oral mucosa lesions and/or any oral habits pertaining to arecanut. The patients were divided into three clinical grades with a diagnosis of oral submucous fibrosis in Group 1, and they were assessed by the following criteria: stages I, II, and III. Colorimetry was utilized to measure the Cu and Zn levels in the tissue and blood of the 40 subjects. An interview focused on oral habits, including chewing areca nut, gutkha (commercially available processed areca nut preparations), and paan (betel quid), as well as the time of consumption and number of intakes of each habit every day for the enrolled subjects. The average levels of Zn and Cu in the tissues of the OSMF group were 25.18 and 4.31 μg/gm, respectively. The average serum Cu concentration and serum Zn concentration in the OSMF group were 1.00 and 0.92 μg/mL, respectively. The Cu levels in the oral submucous fibrosis group were significantly different from those in the control group, whereas the variation in the levels of Zn in the tissue was also significant. The levels of Zn and Cu in the serum did not significantly differ from those in the control group [18]. By measuring only Cu and Zn, the study provides a limited view of trace element changes related to fibrosis and oxidative stress, and other elements such as Fe, Se, or Mn were not addressed.

Low Zn levels in OSMF tissue may indicate local Zn deficiency, which could reduce the oral mucosa’s ability to repair itself and protect against oxidative damage, possibly contributing to fibrosis progression and cancer risk.

Nonetheless, the study does not discuss the mechanism by which Zn depletion occurs in OSMF tissue. It is unclear whether the reduced tissue Zn reflects reduced dietary intake, competitive displacement by elevated Cu, since Cu and Zn compete for intestinal metallothionein binding and intestinal absorption, or active local Zn depletion due to the disease process itself.

A study by Guruprasad et al. [19] examined the connection between Fe and serum vitamin C levels in individuals with OSMF. The amount of Fe in the serum may indicate the progression of the developing illness. OSMF is caused by a disruption in the metabolism of collagen, where vitamin C is essential for the hydroxylation reaction that converts proline into hydroxyproline. This reaction calls for both vitamin C and ferrous Fe. Few studies have investigated the serum levels of vitamin C and its relationship with Fe in patients with OSMF, whereas several have concentrated on micronutrients and other antioxidant levels. Thirty‐five OSMF patients and 50 healthy, habit‐free, deleterious persons (controls) participated in this study. Two milliliters of venous blood was drawn from each subject. The tripyridyl method was used to measure the Fe content in the serum, and the 2–4 dinitrophenylhydrazine method was used to evaluate the vitamin C level in the blood. The Fe and vitamin C levels in the serum were significantly lower in OSMF patients than in controls, according to the results. These results imply that vitamin C and Fe insufficiency may interact with chemical, thermal, and/or mechanical aspects related to areca nut consumption to cause OSMF [19]. The study does not measure tissue levels of either vitamin C or Fe, confining itself entirely to the serum compartment. This is a significant limitation, as changes in serum do not necessarily reflect changes at the tissue level where collagen synthesis actually occurs. The critical biochemical events of OSMF—excessive collagen cross‐linking, lysyl oxidase upregulation, and hydroxylation reactions—occur within the submucosal connective tissue, not in the circulation.

The authors stated that serum Fe content can be a predictor for the progression of this condition, yet the study has no disease staging and no longitudinal follow‐up, conditions that are essential for establishing the progression of the disease.

### 3.4. OLP

Lichen planus is a chronic inflammatory condition affecting the stratified squamous epithelium. The manifestation of this condition primarily occurs in the oral mucosa, and its presence in areas such as the urinary tract, nasal mucosa, larynx, skin, genitalia, nails, hair follicles, esophagus, and sometimes the eyes may occur in nearly 15%–20% of cases [20]. A review of the literature revealed that the following elements, which include Zn, Fe, calcium, aluminum, gallium, and mercury, seem to play a role in OLP. However, since Zn is crucial for epithelial growth, Zn deficiency can trigger the cytotoxic activity of T‐helper 2 cells, a factor that appears to be linked with LLP.

Iijima et al. [21] directly analyzed trace elements in the oral mucosa affected by OLP using the particle‐induced X‐ray emission (PIXE) (2015) method to identify causal metals. There were 72 OLP patients in all, 25 males and 47 women, with an average age of 60.1 years (OLP group). The control group comprised of elemental analysis data from 100 patients with healthy oral mucosa that were collected using the PIXE method. The oral mucosa of the OLP and control groups showed the presence of twelve contaminated elements and seventeen necessary elements. In terms of element predominance in the oral mucosa, the levels of Au and Y in the OLP group were significantly higher than those in the control group, whereas the levels of contaminating elements such as gallium, stibium, aluminum, lead, and mercury were noticeably lower. The contaminating elements in the oral mucosa of the OLP group showed a lower appearance ratio in terms of elemental concentration compared to the control group. That being said, the contaminating elements of the OLP group were more abundant overall. Of the 25 participants in the OLP group and the seven in the control group, identical serum, oral mucosa, and saliva samples were taken. Saliva and serum concentrations differed based on the components. There was a general pattern that showed that while necessary elements were more prevalent in serum than in saliva, contaminating elements tended to be more plentiful in saliva. It is thought that some parts came from outside sources. Aluminum has been found to be prevalent in OLP‐affected mucosal tissues in the past. Because titanium is used in food coloring and aluminum is included in baking powder and related items, consuming too much of these substances may result in the development of OLP [21]. The study does not record the dental restoration status of OLP patients, making it impossible to determine whether the detected contaminants correspond to dental materials present in the oral cavity of those patients. The paper does not specify on how mucosal biopsies were processed for PIXE analysis. Whether samples were freeze‐dried, formalin‐fixed, or subjected to any other preparative procedure is unstated. This matters significantly because formalin fixation can leach diffusible elements from the tissue, introducing systematic underestimation of certain elements.

In their research, Chen et et al. [22] examined whether there was a positive correlation between deficiencies in Fe, hemoglobin (Hb), folic acid, and vitamin B12 and high blood homocysteine levels in patients with OLP. They assessed the blood levels of Fe, vitamin B12, Hb, homocysteine, and folic acid in 352 OLP patients and compared the measurements with the corresponding levels in 352 patients who were of the same age and sex. The findings of their study revealed that 77 patients with OLP had deficiencies in Hb, whereas 48 OLP patients had low Fe levels (<60 μg/dL), 25 OLP patients had low vitamin B12, and one OLP patient had low folic acid levels (<4 ng/mL). Additionally, 52 OLP patients presented comparatively high homocysteine levels in their blood. Compared with those of healthy participants, the incidence rates of Fe, vitamin B12 deficiency, Hb, and increased homocysteine levels in blood were greater in patients with OLP. OLP patients were further categorized into those with major erosive OLP and those with nonerosive OLP. Researchers have reported that the mean homocysteine level in major erosive OLP patients is variably greater than that in nonerosive OLP patients. The average levels of folic acid and vitamin B12 were notably lower among OLP patients than among controls in this study. Researchers have concluded that a significant association exists between OLP and deficiencies in Hb, Fe, folic acid, and vitamin B12, as well as with high blood homocysteine levels. Furthermore, they suspect that a strong interconnection is likely to be present between increased levels of homocysteine in the blood and the severity of OLP [22]. The study does not fully clarify whether hematinic deficiencies are a cause or a consequence of OLP. Inflammatory conditions like OLP can alter Fe levels, reduce dietary intake, and increase micronutrient requirements during ongoing tissue injury and healing. The study does not distinguish between low hematinic levels that may contribute to OLP and those that may result from the disease process.

Ma et al. [23] aimed to evaluate the intracellular Ca2+ concentration, which has a role in lymphocytic activation. They also examined the concentration of oligodendrocyte lineage–myelin basic proteins and stromal interaction molecule 1 in patients with OLP and compared them with controls. In continuation with the lymphocytic activation, the intracellular Ca2+ concentration was markedly lower in OLP subjects than in controls. The possibility of autoimmunity may be the cause of an increase in the lymphocytic infiltration in OLP. The dysfunctional activity of intracellular Ca2+ in the peripheral lymphocytes in subjects with OLP has a role in the progression of this condition [23]. The study only measured blood calcium and not calcium changes in OLP lesions, and it cannot confirm that the findings reflect the affected tissue. The study does not clearly show how changes in cellular calcium levels could contribute to the abnormal T‐cell activity in OLP.

A study undertaken by Bao et al. [24] investigated the hemanitic deficiencies of individuals with OLP and associated them with healthy controls. A total of 236 individuals with OLP and 226 normal subjects who were matched in terms of sex and age were included in the study. The levels of Hb, folate, vitamin B12, and ferritin were compared between patients with OLP and healthy individuals. The study used the reticular, erosive, and ulcerative system for scoring OLP. The incidence of vitamin B12 and serum ferritin deficiency in patients with OLP was notably greater than that in healthy controls. They inferred that insufficient intake of Fe, folate, and vitamin B12 can markedly disrupt immune responses and cell‐mediated immunity [24].

### 3.5. Lichenoid Reaction

Warnakulasuriya et al. [25] suggested that ample evidence indicates an increased risk of oral cancer in patients diagnosed with “oral lichenoid lesions” (OLLs). Oral lesions with lichenoid characteristics but without the typical histopathological or clinical features of OLP may present asymmetrically or result from reactions to dental restorations or drug‐induced causes [25]. OLLs exhibit lichen‐like reactions and share similar clinical and histopathological features with OLP. Analyzing trace metals in OLLs could aid in distinguishing symptoms and identifying metallic restorations for their occurrence [26]. The literature extensively documents that biological processes such as dissolution, evaporation, corrosion, or other forms of degradation result in the release of mercury from amalgam alloys. The mercury released is absorbed by the oral soft tissues, which causes toxic or allergic reactions in sensitized individuals manifesting as lichenoid reactions [27]. Metals such as nickel, gold, palladium, chromium, mercury, cobalt, or Cu have been identified as potential factors for lichenoid reactions [28].

Pezelj‐Ribarić et al. [29] evaluated the association between amalgam‐based restorations and oral lichenoid reactions (OLRs). Twenty individuals with OLRs and 20 normal subjects who underwent surgery between 2001 and 2005 were included in the study. Patients underwent patch testing of the skin performed by a physician. Sensitization to inorganic mercury or amalgam was observed in 16 patients out of 20. The restoration was replaced with filling‐based composite resin. Complete healing was achieved in 16 patients, whereas three patients achieved marked improvement; no improvement was observed in one patient. According to clinical observations, restorative therapy promoted tissue healing in these patients [29]. The study investigated the relationship between OLRs and dental amalgam restorations, with particular interest in immunological responses after amalgam replacement. However, it is important to note that the study did not directly quantify trace elemental levels (such as mercury, silver, tin, Cu, Zn, or other metals) in saliva, blood, tissue, or restorations.

Rasul et al. [30] evaluated longstanding OLRs in a patient receiving gold dental implants. However, according to recent literature, this change is believed to be a changed immune reaction marked by irregular T‐cell activation and consequent inflammation. This reaction can be linked to conditions such as allergic contact dermatitis and hepatitis. Gold‐induced lichenoid reactions have been identified as a consequence of the prolonged use of gold salts or supplements containing gold [30].

Sensitization to palladium is gaining much attention, as the prevalence of this contact allergy to this metal has been significantly underestimated. Muris et al. [31] evaluated whether sensitization to palladium or nickel is correlated with various clinical factors, including exposure to different types of dental alloys and oral symptoms or complaints. They assessed the interaction between the dental alloys and observed clinical indications of oral disease, specifically OLRs. These alloys are categorized into dental amalgams, dental crowns, and prostheses. In all the patients, increased sensitization to palladium and nickel was observed, with xerostomia and dry mouth. Oral exposure to palladium may affect the threshold level for nickel stimulation. Nickel is sensitive to changes in pH, which can result in greater ion release under acidic conditions, which explains the possible observations [31].

### 3.6. Role of Elements in Oral Malignancies

In areas where tobacco‐related practices are still prominent, oral cancer is more common than any other cancer‐related illness or mortality worldwide. Important components serve as microsources for vital elements in biochemical processes, and new research indicates that the development and spread of cancer may affect the amounts of these components [14]. SCC, which affects individuals 50–70 years of age who mostly consume tobacco and alcohol, accounts for 90% of oral cancer cases and can be associated with infection from the human papillomavirus, genetic predisposition, and passive smoking exposure. Tobacco can increase DNA damage, as smoke itself comprises numerous identified toxic and carcinogenic compounds, including inorganic compounds such as chromium, Fe, magnesium, Mn, nickel, potassium, arsenic, calcium, chlorine, cobalt, Cu, and Zn [32].

In 2006, Khanna and Karjodkar [33] evaluated the levels of trace elements (Fe, Cu, and Se) and circulating immune complexes (CICs) in the blood of patients with oral squamous cell carcinoma, OLK, and OSMF. Examining changes in these measures and finding trustworthy indicators of the onset and course of the disease were the goals of this investigation. Using serum precipitation with 37.5% polyethylene glycol (PEG) 6000, CICs were quantified. The colorimetric dipyridyl method, differential pulse cathodic stripping voltammetry, and oxalyl dihydrazide method were used to evaluate the serum levels of Cu, Fe, and Se, respectively. The data analysis indicated that patients with cancer and precancer had increased amounts of CICs. Serum Fe levels considerably decreased in the cancer group, whereas serum Cu levels progressively increased from precancer patients to cancer patients. In the cancer group, the concentration of Se decreased significantly. The best indicators of the likelihood of lesions among CICs are serum Cu, Fe, and Se. These are followed by age, serum Fe, CICs, and serum Se. The results of this study imply that the pathophysiology and progression of oral premalignant and malignant lesions may be related to these biological and immunological markers. As a result, some actions might be helpful in early detection, management, and assessment of the effectiveness of treatment in such circumstances [33]. The increase in serum Cu is an important observation; however, the study cannot establish that Cu elevation directly contributes to malignant transformation. Although Fe is essential for many biological processes, the study did not determine whether Fe deficiency preceded cancer development or occurred as a consequence of cancer progression. The study also does not demonstrate the accumulation of trace elements within oral lesions or prove that these alterations initiate cancer. Recently, reports have indicated that Mn can enhance the antitumor immune response through the cGAS‐STING pathway [34]. The concentration of Mg and its correlation with various types of cancer, particularly regarding factors such as the presence of electrolytes in foods in the daily diet, should be taken into consideration. In this regard, research has generally shown the positive impact of magnesium consumption on cancer [35]. Chromium has been considered to initiate carcinogenesis by producing ROS and suppressing the p53 gene [36].

An investigation conducted by Dziewulska et al. [37] aimed to identify potential markers that could assist in the diagnosis of oral cancer, and they estimated the mineral composition of saliva in individuals with oral cancer. A total of 34 patients between 35 and 72 years of age who were confirmed to have a diagnosis of oral cancer, which included seven women and 27 men, were included in the study prior to the initiation of treatment. Unstimulated saliva was collected in containers. The concentrations of calcium, magnesium, Fe, and phosphorus were estimated via colorimetric methods, whereas the concentrations of sodium and potassium were estimated via ion‐selective electrodes. The study and control groups were closely monitored, notable variations were observed, and the concentration of sodium was elevated in the study group. Among the various sites of cancer, cancer affecting the floor of the oral cavity had the highest concentration of salivary sodium, and cancer of the tongue or parotid gland had the lowest levels, whereas the calcium levels were highest in cancer in the floor of the oral cavity, in patients with cancer involving the floor of the oral cavity, and low in patients with tongue cancer [37]. The authors in this study assessed magnesium because of its biological importance in enzyme activity and cellular metabolism; therefore, differences were noted according to tumor location, but magnesium did not demonstrate clear diagnostic usefulness. The study also provides useful preliminary evidence for exploring saliva as a diagnostic medium, but the measured minerals (Na, K, Ca, Mg, Fe, and P) cannot be considered confirmed oral cancer biomarkers without larger longitudinal studies and direct analysis of tumor‐associated elemental changes.

## 4. Discussion

Trace elements have a major role in various physiological processes in the human body. Any alteration in these elemental concentrations can lead to pathological changes. Table 1 summarizes various physiological roles of trace elements and how their concentrations are altered in pathological conditions.

**Table 1:** Physiological and pathological roles of trace elements.

Element	Physiological	Pathological
Zn	Zinc helps protect cells from harmful reactive oxygen species and bacterial toxins by increasing the antioxidant activity of cysteine‐rich metallothionein proteins [38]	Imbalance in zinc levels can disrupt normal cell metabolism and may contribute to disease development, including tumor formation [39]
Cu	Copper can exist in different forms (Cu^+^ and Cu^2+^), allowing it to support important cellular functions such as enzyme activity, energy production, iron absorption, antioxidant defense, and connective tissue formation [40]	Cancer cells require more copper to support their rapid growth and division. Copper promotes the formation of new blood vessels by activating factors such as VEGF, helping tumors receive nutrients and continue growing [41]
Se	Selenium helps normal metabolism and helps control inflammation through its anti‐inflammatory effects [42]	Selenite is converted in the body to form selenocysteine and selenium‐containing proteins. Lower selenium levels in oral cancer patients may result from reduced selenium availability in the blood [43].
Fe	The change between Fe^2+^ and Fe^3+^ states of iron plays an important role in catalyzing and supporting various biological reactions [44]	Hydroxyl radicals and iron‐related reactive compounds can harm DNA by causing changes and breaks in its structure [44]
Ca	Calcium acts as an important intracellular messenger that regulates various cellular functions, including programmed cell death (apoptosis) [45]	In cancer cells, abnormal calcium signaling may lead to altered CAMK II activation. Changes in the expression and function of Fas can also affect its role in regulating apoptosis
Mg	Magnesium is an essential mineral involved in energy production, DNA repair, and maintaining genetic stability. In normal cells, magnesium helps protect DNA and reduces the risk of mutations [46]	In cancer cells, low magnesium levels may contribute to tumor development, while adequate magnesium levels can help limit tumor growth and support immune function [46]
Mn	Manganese is a crucial helper molecule for enzymes, especially manganese superoxide dismutase (MnSOD), which protects cells by neutralizing harmful reactive oxygen species (ROS) in the mitochondria [47]	It can help prevent tumor growth by increasing oxidative stress and activating immune responses, but in certain conditions, it may also contribute to cancer spread (metastasis) [47]

Although numerous studies have demonstrated significant associations between altered trace element concentrations and OPMDs or oral squamous cell carcinoma, these findings should not be interpreted as evidence of direct causality. The majority of the available literature consists of observational studies, which are inherently limited in establishing cause‐and‐effect relationships. Altered trace element profiles may represent the consequences of chronic inflammation, oxidative stress, nutritional deficiencies, altered metabolism, tissue remodeling, or tumor‐associated physiological changes rather than primary drivers of carcinogenesis. Furthermore, bidirectional interactions are possible, whereby disease progression itself influences trace element homeostasis. Consequently, the biological mechanisms discussed in this review should be regarded as plausible hypotheses supported by experimental and mechanistic evidence rather than definitive causal pathways. Future longitudinal, mechanistic, and interventional studies are required to clarify the temporal and causal relationships between trace element dysregulation and oral carcinogenesis.

The extensive evidence reviewed over the past two decades strongly supports the concept that systemic and local alterations in trace element metabolism are actively involved in the etiopathology and progression of OPMDs and oral squamous cell carcinoma (OSCC), positioning them as valuable biomarkers. Cu is consistently implicated, particularly in conditions driven by tobacco and areca nuts. Its proangiogenic activity and role as a prooxidant, stimulating ROS production, drive carcinogenesis, which is reflected in the progressive increase in serum Cu levels observed from OLK to established OSCC [6, 33]. In oral submucosal fibrosis, the pathology is linked to localized tissue Cu, which acts as a crucial cofactor for lysyl oxidase, promoting collagen cross‐linking and fibrogenesis [10, 18]. Conversely, Zn often plays a protective role as an antioxidant and immune modulator. Deficiencies in serum or plasma Zn are observed in OLK and are correlated with the severe erosive subtype of OLP, suggesting a breakdown in the body’s antioxidant defense and immune–epithelial balance [8, 21]. Consequently, the Cu‐to‐Zn ratio is emerging as a more powerful and reliable diagnostic and prognostic indicator [9]. Deficiencies in Fe, which is frequently coupled with low vitamin B12 and folic acid contents, are prevalent in both OSMF and OLP patients, underscoring the link between systemic nutritional status and the severity or progression of oral mucosal disease [11, 23, 24]. Furthermore, the substantial decrease in Se observed specifically in the OSCC group suggests a severe loss of antioxidant capacity as malignancy takes hold, making Se a potential prognostic marker [37]. Mn is a trace element for physiological functions, and Mn2+ is the most prevalent form observed in the human body [48]. Elemental analysis of saliva also has diagnostic potential, with elevated sodium levels noted in oral cancer patients, particularly those with floor of the mouth lesions [25]. Finally, the distinct etiology of OLRs is strongly linked to localized hypersensitivity reactions caused by the release and absorption of metals—including mercury, gold, palladium, and nickel—from dental restorations [29, 31, 32]. This distinction highlights the clinical utility of elemental analysis for differential diagnosis and the effectiveness of restoration replacement in managing OLRs [30]. Collectively, these findings necessitate the integration of trace element screening into advanced diagnostic, nutritional intervention, and preventive strategies within oral healthcare. Table 2 summarizes the roles of trace elements in various OPMDs and oral cancer.

**Table 2:** Summary of findings showing the levels of various trace elements in potentially oral malignant disorders and oral cancer.

Author and year	Study design	Number of participants (control)	Study	Element assessment	Technique employed	Inferences
Mampilly et al. (2021) [6]	Comparative	20	10 (OL) 10 (OSCC)	Elevated ceruloplasmin and reduced selenium	Atomic absorption spectrometer	Serum levels of ceruloplasmin and selenium may be useful as biomarkers for detecting and monitoring leukoplakia and squamous cell carcinoma [6]
Sourabh et al. (2023) [7]	Comparative cross‐sectional observational study	20	30 (OL) 30 (OSCC)	Elevated copper	Atomic absorption spectrometer	Areca nuts contain high levels of copper and are considered a major contributing factor in precancerous oral conditions such as oral submucous fibrosis and leukoplakia [7]
Nayak et al. (2011) [18]	Case–control observational study	20	20 (0SMF)	Elevated copper and zinc	Colorimetric method	Copper released during areca nut chewing comes into direct contact with oral mucosal cells and may contribute to the initiation and development of oral submucous fibrosis (OSMF) [18]
Ma et al. (2016) [23]	Case–control observational study	16	39 (OL)	Reduced ionized and serum calcium	Quantitative real‐time PCR and Western blot	Patients with oral lichen planus (OLP) have lower intracellular Ca^2+^ levels compared with healthy individuals, suggesting abnormal calcium signaling. Increased expression of Golli‐MBP and STIM1 may contribute to the development of OLP [23]
Rasul et al. (2022) [30]	Case report	—	1	Elevated gold	Exposure to metal	Prolonged exposure to gold from dental implants may cause an abnormal immune reaction, leading to T‐cell activation, inflammation, and oral lichenoid lesions [30]
Khanna and Karjodkar (2006) [33]	Randomized control clinical trail	30	30 (OSMF) 30 (OSCC)	Reduced iron and elevated copper	Colorimetric oxalyl dihydrazide method	Changes in trace element levels and immune responses are linked to the development and progression of precancerous oral lesions and oral cancer
Tadakamadla et al. (2011) [49]	Case–control observational study	50	50	Elevated copper and reduced iron	Atomic absorption spectrometer	High copper levels in areca nuts, a major cause of OSMF, may promote fibrosis by increasing lysyl oxidase activity, which enhances collagen formation and reduces collagen breakdown, leading to collagen accumulation. Iron is also used in collagen synthesis for hydroxylation of proline and lysine, resulting in decreased serum iron levels in OSMF patients
Shetty et al. [50]	Case–control observational study	50	50 (OL) 50 (OSMF) 50 (OSCC)	Elevated copper and reduced zinc	GBC Avanta atom absorption spectrophotometer	Copper plays an important role in cell metabolism as a component of enzymes such as tyrosinase, uricase, and cytochrome oxidase, which help regulate oxidation reactions

Laser‐induced breakdown spectroscopy (LIBS) is a qualitative/semiquantitative elemental characterization technique based on atomic emissions from samples [51]. It can analyze materials from a distance, without needing sample preparation, and provides results in a very short period of time. LIBS provides excellent elemental imaging and finds applications across a range of fields, including in industrial processes, environmental, and biomedical analyses [52]. AAS is a widely used technique in analytical chemistry because it can detect many metals and metalloids with good sensitivity and usually gives accurate results with fewer interferences. AAS determines element concentrations by measuring how atoms absorb specific wavelengths of light, providing accurate and sensitive results, especially for metals and trace elements. Both techniques are widely used for detecting small amounts of elements in environmental, biological, food, and industrial samples [53].

Apart from cancer and OMPDs, elemental levels can be influenced by numerous factors such as dietary intake, nutritional deficiencies, systemic diseases, chronic inflammatory conditions, medication use, renal or hepatic dysfunction, geographic and environmental exposure, and occupational hazards.

These may alter trace element levels in serum, saliva, or tissues. These factors contribute to the considerable variations observed across studies and may partially explain inconsistent findings for several trace elements. Future investigations should adopt standardized protocols, carefully characterize study populations, and adjust for these potential confounders to improve the reliability, reproducibility, and clinical interpretation of trace element levels as potential biomarkers.

An additional challenge in translating trace element biomarkers into routine clinical practice is the significant methodological variability among biological specimens and analytical techniques.

Trace element concentrations differ considerably depending on whether serum, saliva, or tissue samples are analyzed, as each specimen reflects distinct physiological and pathological processes. Analytical techniques such as AAS, inductively coupled plasma mass spectrometry (ICP‐MS), LIBS, EDX, and PIXE differ in their sensitivity, specificity, and detection limits. These methodological differences contribute to interstudy and interlaboratory variability, making direct comparison of published results difficult. Importantly, there are currently no universally accepted protocols for specimen collection, processing, analytical calibration, or quality assurance, nor are there clinically validated reference ranges or diagnostic cut‐off values for trace element concentrations in OPMDs or oral squamous cell carcinoma. Consequently, although trace element analysis shows considerable promise as an adjunctive biomarker, harmonization of laboratory methodologies, external quality‐control programs, multicenter validation studies, and establishment of standardized reference intervals are essential before these biomarkers can be reliably translated into routine clinical diagnostics or population‐based screening programs.

Public misconceptions and lack of awareness and understanding of the harmful effects of areca nuts and the importance of cessation programs have been identified as obstacles to areca nut cessation [54].

Studies have shown that habitual arecanut users often show altered levels of trace elements such as Cu, Zn, Fe, and Se in their blood, saliva, and tissue samples compared to healthy individuals. Cu, in particular, has drawn attention because arecanut contains Cu salts that activate lysyl oxidase, an enzyme linked to abnormal collagen cross‐linking and fibrosis in the oral mucosa [55].

From a public health perspective, the potential application of trace element analysis extends beyond individual patient diagnosis to population‐level risk stratification and prevention. In regions with a high burden of tobacco and areca nut use, integrating trace element assessment into existing oral cancer screening initiatives could help identify individuals at increased biological risk when used alongside conventional oral examination. However, several challenges must be addressed before such an approach can be implemented. These include the need for standardized laboratory protocols, the establishment of clinically validated reference ranges, quality assurance across laboratories, cost‐effectiveness analyses, and appropriate training of healthcare personnel. Rather than replacing current screening strategies, trace element analysis may serve as an adjunctive tool within risk‐based screening models, particularly in high‐risk populations. Future implementation research should evaluate the feasibility, acceptability, economic implications, and health‐system integration of such approaches to determine their potential contribution to oral cancer prevention programs and evidence‐informed public health policy.

Measuring these trace elements through techniques such as AAS or ICP‐MS gives us a simple, measurable way to assess disease severity and risk of progression to oral cancer. From a public health perspective, this kind of analysis becomes especially valuable when integrated into mass screening programs in regions where arecanut chewing is common, such as parts of South and Southeast Asia. By combining clinical oral examination with trace element testing, screening camps can identify high‐risk individuals earlier, even before visible lesions become severe, allowing for timely counseling, habit cessation support, and referral for biopsy if needed. This approach makes population‐level screening more effective, as it adds an objective biological marker to the visual assessment typically used in community health camps, ultimately helping reduce the burden of oral cancer in high‐risk populations [56].

## 5. Conclusion

Elements are formed in various forms in nature and are vital for diverse cellular functions at the biological, chemical, and molecular levels. The levels of these trace elements dictate the presence, absence, and progression of various pathological processes. Although they account for only ~0.02% of the total body weight, they play a major role as active centers of enzymes or as trace bioactive substances.

Our literature review revealed that trace elements play a significant role in the pathogenesis of various potentially malignant oral disorders. Cu, Zn, ceruloplasmin, Fe, Mn, Se, and glutathione have been implicated in the causes of L; Hb, Fe, vitamin B12, folic acid, calcium, Zn, aluminum, gallium, mercury, and lead are involved primarily in Lichen planus, whereas Cu, niobium, silver, tantalum, Zn, Fe, Hb, and calcium appear to play significant roles in oral submucous fibrosis. Since these essential elements are crucial in various biochemical processes of the body, multiple studies have demonstrated their associations with oral malignancies, particularly the roles of Cu, Fe, Se, sodium, calcium, magnesium, Zn, and potassium in the pathogenesis of oral squamous cell carcinoma. These findings suggest that the analysis of trace elements has a promising potential as an adjunct biomarker for the detection, risk assessment, and monitoring of disease. However, the current evidence is derived mainly from observational studies. Considering that major limitations remain such as the absence of validated clinical protocols and well‐documented longitudinal studies, trace elemental analysis should be regarded as an emerging research tool rather than a clinical diagnostic tool at the present time. Large‐scale prospective studies in the future should be planned along with standardization of analytical techniques before these elemental biomarkers can be incorporated as a clinical diagnostic tool or as a screening tool in public health programs.

Translating such evidence into public health action can enable policymakers to identify at‐risk populations, promote region‐specific nutritional interventions, and integrate elemental screening into community‐based oral health programs.

## Author Contributions

Conceptualization, data curation, visualization: Hannah Haneef and Ravikiran Ongole. Formal analysis: Hannah Haneef, Ravikiran Ongole, Sara J. Ommen, Joanna Baptist, and Prasanna Mithra. Investigation: Hannah Haneef. Methodology: Hannah Haneef, Sara J. Ommen, and Joanna Baptist. Project admin: Ravikiran Ongole and Prasanna Mithra. Resources: Hannah Haneef, Sara J. Ommen, Joanna Baptist, and Ravikiran Ongole. Supervision: Ravikiran Ongole. Validation: Hannah Haneef, Joanna Baptist, Ravikiran Ongole, and Prasanna Mithra. Writing – original draft: Hannah Haneef, Ravikiran Ongole, Joanna Baptist, and Sara J. Ommen. Review and editing: Joanna Baptist, Ravikiran Ongole, Hannah Haneef, Sara J. Ommen, and Prasanna Mithra.

## Funding

This study did not receive any specific funding.

## Disclosure

All authors have read and approved the final version of the manuscript. All authors had full access to all of the data in this study and take complete responsibility for the integrity of the data and the accuracy of the data analysis.

## Conflicts of Interest

The authors declare no conflicts of interest.

## Data Availability

The data that support the findings of this study are available from Joanna Baptist (corresponding author) upon reasonable request.
